# Sampling-free investigation of microbial carbon source preferences on renewable feedstocks via online monitoring of oxygen transfer rate

**DOI:** 10.1007/s00449-024-03117-x

**Published:** 2024-12-16

**Authors:** Luca Antonia Grebe, Paul Richter, Torben Altenkirch, Marcel Mann, Markus Jan Müller, Jochen Büchs, Jørgen Barsett Magnus

**Affiliations:** 1 AVT–Biochemical Engineering, RWTH Aachen University, Forckenbeckstraße 51, 52074 Aachen, Germany; 2 Bioeconomy Science Center (BioSC), 52425 Jülich, Germany

**Keywords:** Carbon source screening, Complex feedstock, *Ustilago* sp., *Escherichia coli*, Diauxic growth, Polyauxic growth

## Abstract

The transition towards sustainable bioprocesses requires renewable feedstocks to reduce dependency on finite resources. While plant-based feedstocks offer significant potential, their complex composition poses new challenges. The microorganisms often exhibit polyauxic growth when presented with multiple carbon sources simultaneously, consuming them in a distinct order according to their carbon source preferences. The traditional investigation of polyauxic growth involves laborious sampling and offline analysis, hindering high-throughput screenings. This study introduces an efficient method for identifying carbon source consumption and their order of metabolization by various microorganisms using the respiration activity monitoring system (RAMOS) in shake flasks. As aerobic carbon metabolization and oxygen consumption are strictly correlated, the characteristic phases of polyauxic growth are visible in the oxygen transfer rate (OTR) and can be assigned to the respective carbon sources. An extended 16-flask RAMOS enables real-time monitoring of microbial respiration on up to seven carbon sources and one reference cultivation simultaneously, thus providing crucial insights into their metabolization without extensive sampling and offline analysis. The method’s accuracy was validated against traditional high-performance liquid chromatography (HPLC). Its applicability to both fast-growing *Escherichia coli* (investigated carbon sources: glucose, arabinose, sorbitol, xylose, and glycerol) and slow-growing *Ustilago trichophora* (glucose, glycerol, xylose, sorbitol, rhamnose, galacturonic acid, and lactic acid) was demonstrated. Additionally, it was successfully applied to the plant-based second-generation feedstock corn leaf hydrolysate, revealing the bioavailability of the included carbon sources (glucose, sucrose, arabinose, xylose, and galactose) and their order of metabolization by *Ustilago maydis*.

**Supplementary Information:**

The online version contains supplementary material available at 10.1007/s00449-024-03117-x.

## Introduction

Considering the environmental impacts associated with current production and consumption patterns, using renewable raw materials has never been more urgent. Current industry, heavily reliant on fossil-based resources, contributes significantly to greenhouse gas emissions [1]. In biotechnology, the necessary transition towards sustainability is often accomplished by using a variety of different feedstocks, often derived from various waste streams of different industries. The valorization of these waste streams not only reduces dependency on finite resources but also assists in the management of agricultural waste, thereby supporting environmental sustainability [2, 3].

The large number of different potential feedstocks offers immense potential but also comes with challenges. Most of these feedstocks consist of many different carbon sources, which are present in different concentrations depending on the source. For the sake of simplicity, this study focuses on plant-based feedstocks and glycerol, which is available in large quantities as a waste product from biodiesel production. The plant-based feedstocks are inherently complex, containing a diverse range of saccharides such as glucose, xylose, fructose, arabinose, galactose, sucrose, and different organic acids like acetate, galacturonic acid or glucuronic acid. Furthermore, the plant-based feedstocks also contain sugar alcohols like sorbitol and insoluble fractions of cellulose, and lignin [4, 5]. To be able to convert this complex mixture of various carbon sources into a product of interest, the metabolic potential of various organisms has to be harnessed. The capacity of microorganisms to metabolize carbon sources varies considerably, influenced by the specific organism and its metabolic capabilities. Each organism shows different carbon source preferences and varying growth rates on every substrate [6, 7]. This behavior was first demonstrated by Monod [8], showing that microorganisms can exhibit diauxic growth when presented with two carbon sources in excess. This phenomenon is marked by bi-phasic exponential growth intermitted by a lag-phase of minimal growth, which results from the buildup time of different enzymes to metabolize the new carbon source [9]. When three or more carbon sources are provided, the polyauxic growth can be observed.

To elaborate the preferences for individual carbon sources and the resulting order of metabolization, traditionally, a labor-intensive and time-consuming process is required. This involves cultivating the organism on a mixture of the pure carbon sources, followed by sequential sampling and offline analysis to determine the carbon source consumption, typically using HPLC [7]. This method, while effective, is inefficient and limited in its throughput, which hinders high-throughput screenings. Furthermore, to estimate the order of metabolization, a comparison of the maximum growth rate on the individual pure carbon source is often used. It is particularly striking that carbon source mixtures from complex feedstocks are rarely evaluated, as the amount of work involved would be much higher than cultivation on pure substances. Given these challenges, the presented research introduces a novel method that significantly enhances the efficiency of evaluating carbon source utilization by different microorganisms on mixtures of pure or complex carbon sources by eliminating the need for extensive sampling and offline analysis. The core of the approach is the online monitoring of the oxygen transfer rate (OTR) of parallelized cultivations using the respiration activity monitoring system (RAMOS) in shake flasks. In aerobic cultivations, oxygen consumption strictly correlates with carbon metabolism and distinct phases of diauxic growth are observable in the OTR [10, 21, 22]. However, these phases previously could not be assigned to the respective carbon sources based solely on the OTR data. The presented method addresses this limitation by parallel spiking of the investigated carbon sources, enabling real-time, high throughput monitoring of polyauxic growth and providing crucial insights into carbon source metabolization [10]. By enabling a more efficient assessment of renewable feedstocks, the developed method supports the enhancement of bioprocesses that are both environmentally friendly and economically viable. This is particularly relevant in the context of industries trying to reduce their carbon footprint and transition towards a circular economy.

## Materials and methods

### Microorganisms

The experiments were performed with the strains *Escherichia* *coli* BL23(DE3) [10, 11], *Ustilago trichophora* TZ1 [12] and *Ustilago maydis* MB215Δcyp1Δemt1 [13].

### Cultivation conditions and media

All shake flask cultivations were performed in unbaffled 250 mL shake flasks with a filling volume (V_L_) of 10 mL, a shaking frequency (n) of 350 rpm, and a shaking diameter (d_0_) of 50 mm (Climo-Shaker ISF1-X, Adolf Kühner AG, Birsfelden, Switzerland). The potential for unavoidable evaporation phenomena during shake flask cultivation is considered to be negligible due to the relatively short cultivation times and low cultivation temperatures employed.

The experiments with *E. coli* were performed at 37 °C and 30 °C in a modified version of Wilms & Reuss synthetic medium (referred to as Wilms-MOPS medium) [14, 15]. The medium consists of 6.98 g/L (NH_4_)_2_SO_4_, 3 g/L K_2_HPO_4_, 2 g/L Na_2_SO_4_, 0.5 g/L MgSO_4_ ∙ 7 H_2_O, 41.85 g/L (0.2 M) 3-(N-morpholino)-propanesulfonic acid (MOPS), 0.01 g/L thiamine hydrochloride, 0.1 g/L ampicillin and 1 mL/L trace element solution (TES) (0.54 g/L ZnSO_4_ ∙ 7 H_2_O, 0.48 g/L CuSO_4_ ∙ 5 H_2_O, 0.3 g/L MnSO_4_ ∙ H_2_O, 0.54 g/L CoCl_2_ ∙ 6 H_2_O, 41.76 g/L FeCl_3_ ∙ 6 H_2_O, 1.98 g/L CaCl_2_ ∙ 2 H_2_O, 33.39 g/L Na_2_EDTA (Titriplex III)). For the reference cultivation, 5 g/L or 2.5 g/L of glucose, arabinose, xylose, sorbitol, and glycerol was added. The same amount of the individual sugars was additionally supplemented in the respective screening cultivations. The pH of the medium was adjusted to 7.5 using sodium hydroxide and the whole medium was sterilized by filtration. Ampicillin and thiamin hydrochloride were stored at − 20 °C and the trace element solution was stored at 4 °C. All other components were stored at room temperature. The precultures were inoculated from cryo-cultures to an optical density (OD_600_) of 0.1 and harvested during exponential growth. The main cultures were inoculated to an OD_600_ of 0.5 directly from the preculture.

The experiments with *U. trichophora* were performed at 25 °C. Adapted versions of the Verduyn medium according to Geiser et al*.* were used for the experiments [16, 17]. For the experiments characterizing the polyauxie of the carbon source, the medium consisted of 10 g/L NH_4_Cl, 4 g/L KH_2_PO_4_, 0.4 g/L MgSO_4_ ∙ 7 H_2_O, 0.01 g/L FeSO_4_ ∙ 7 H_2_O, 1 mL/L TES and 0.3 M 2-(N-morpholino)ethanesulfonic acid (MES) buffer. The TES contained 15 g/L ethylenediaminetetraacetic acid (EDTA), 4.5 g/L ZnSO_4_ ∙ 7 H_2_O, 0.84 g/L MnCl_2_ ∙ 2 H_2_O, 0.3 g/L CoCl_2_ ∙ 6 H_2_O, 0.3 g/L CuSO_4_ ∙ 5 H_2_O, 0.4 g/L Na_2_MoO_4_ ∙ 2 H_2_O, 4.5 g/L CaCl_2_ ∙ 2 H_2_O, 3 g/L FeSO_4_ ∙ 7 H_2_O, 1 g/L H_3_BO_3_, 0.1 g/L KI. Glucose, glycerol, xylose, sorbitol, rhamnose, galacturonic acid, and lactic acid were added to a concentration of 5 g/L each, if not specified otherwise. The stock solutions for the carbon sources, NH_4_Cl, NaNO_3_, KH_2_SO_4_, and MgSO_4_ were prepared separately and sterilized by autoclaving. The pH value of the KH_2_PO_4_ solution was adjusted to 6.5 with 10 M NaOH. FeSO_4_, TES, and MES were sterilized via sterile filtration. The pH value of the MES buffer was adjusted to 7.2 with NaOH pellets. Stock solutions of FeSO_4_ and TES were stored at 4 °C. All other components were stored at room temperature. The preculture medium consisted of 50 g/L sucrose, 4 g/L NH_4_Cl, 2 g/L KH_2_PO_4_, 0.4 g/L MgSO_4_ ∙ 7 H_2_O, 0.01 g/L FeSO_4_ ∙ 7 H_2_O, 1 mL/L TES and 0.1 M MES buffer [18]. The precultures were inoculated from cryo-cultures to an optical density (OD_600_) of 0.1 and harvested during exponential growth. The main cultures were inoculated to an OD_600_ of 0.3 directly from the preculture.

The experiments with *U.* *maydis* were also performed at 25 °C. Again, the same adapted version of the Verduyn medium was used for the experiments (see *U. trichophora* experiments). Unlike the medium used for the *U. trichophora* experiments, no carbon source was added to the reference medium. Instead, the water content of the medium was partially replaced by corn leaf hydrolysate of 12 w/v %. The reference as well as all other cultivations were substituted with 45 v/v % of the hydrolysate. The tested carbon sources were then added in individual batches and the volume was equalized with the remaining distilled water. For the hydrolyzation of the corn leaves, the hydrolyzation protocol from Rodrigues et al. established in 2015 was used [19]. This protocol features a hydrolyzation in 0.1 M sodium acetate buffer, therefore, small amounts of acetate (0.045 M) will be transferred into the cultivation medium.

### Sampling and offline analytics

The samples were drawn at the end of the cultivation from the respective RAMOS flasks. The samples during the cultivation were drawn from offline flasks cultivated in parallel under the same conditions. For measurements of the OD_600_, culture broth was diluted with 9 g/L NaCl in a range of 0.1–0.4 and measured with a spectrophotometer.

To quantify the tested carbon sources, samples were analyzed via high performance liquid chromatography (HPLC). HPLC samples from shake flasks were prepared via centrifugation of 1 mL each at 18,000 g for 5 min and subsequent sterile filtration. A Thermo Fisher Ultimate 3000 (Thermo Fisher Scientific (Waltham, Massachusetts, United States) combined with a Rezex ROA-Organic Acid H + (8%) LC Column 300 × 7.8 mm (Phenomenex, Inc., Torrance (CA), United States) and a RefractoMax 520 RI detector (Thermo Fisher Scientific, Waltham (Massachusetts), United States) was used. As a mobile phase 25 mM sulfuric acid with a flow velocity of 0.8 mL/min at 75 °C was used.

### Characterization of carbon source metabolization via online monitoring of the OTR

**Fig. 1:**
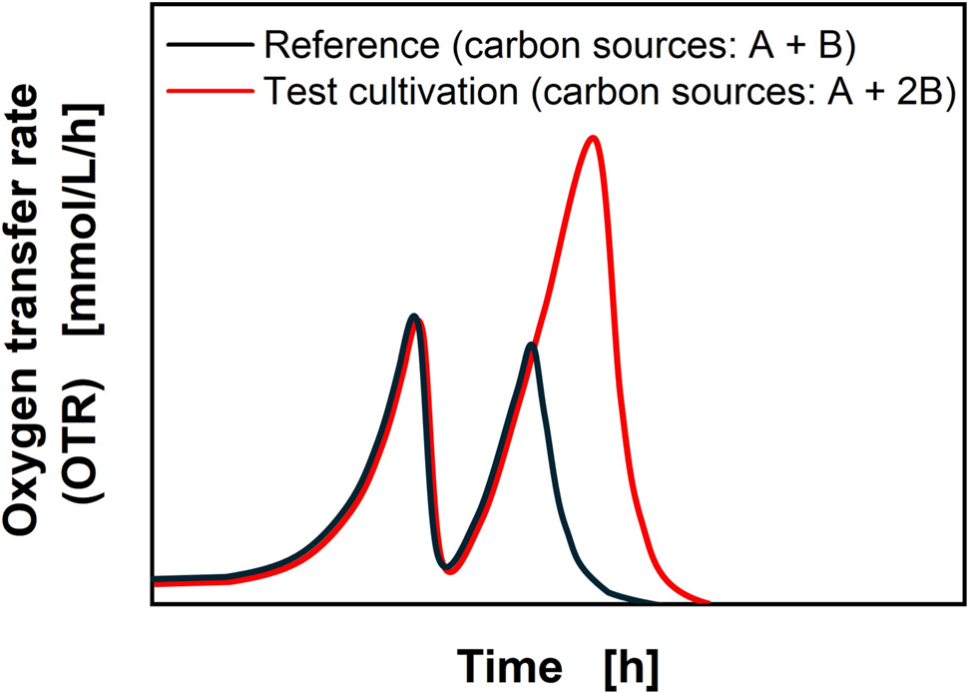
Schematic visualization of the developed method. Characteristic OTR of diauxic growth on complex carbon source mixture (Reference) and effect of additional amount of the investigated carbon source (Test cultivation)

Carbon metabolism is inherently linked to oxygen consumption in aerobic cultivations. The resulting oxygen uptake rate can be regarded as equal to the OTR if the change of dissolved oxygen concentration in the liquid over time is sufficiently small [20]. Hence, the characteristic phases of diauxic growth during batch cultivations are visible in the OTR, which can be monitored using the RAMOS [21–23]. However, generally, no information about the sequence of carbon source consumption can be drawn from the OTR data alone. Therefore, the identification of the OTR peaks and the assignment to the respective carbon source are usually done by HPLC measurement [23]. For cultivations exhibiting diauxic growth, the supplementation of additional amounts of a single carbon to the medium prolongs the respective growth phase, without influencing the growth on the other carbon source [8]. By comparing this to the reference, the peaks can be identified (Fig. 1).

Plant-based second generation feedstocks generally contain multiple carbon sources [24]. However, due to the complexity of microbial growth, only marginal effects on the OTR might be visible, when an organism is exposed to multiple carbon sources. Therefore, high resolution and reproducibility of biological replicates are crucial to reliably identify deviations from the reference. When using the established 8-flask RAMOS, polyauxic growth on three carbon sources can simultaneously be investigated in biological duplicates. Performing two experiments consecutively can increase the number of investigated carbon sources. However, already slight deviations from the reference, e.g., due to a different preculture, may lead to inconclusive data. By measuring the reference in each experimental run to ensure the reproducibility of both runs, polyauxie of six carbon sources can be investigated [25]. A prerequisite for the presented method is an unlimited growing batch culture. No secondary influence on the OTR should be present. Induction or production processes in general should be avoided, as strong metabolic burdens could be present in the cultivation and do not allow the OTR to be used to analyze the sequence of metabolization of the added carbon sources [26]. In addition, the method presented here only works in batch mode, as no polyauxic behavior would be visible in fed-batch or continuous cultivation. In fed-batch and continuous cultivation, very low substrate concentrations are used to reduce substrate inhibition or loss. Carbon sources that would be metabolized sequentially in excess can be metabolized simultaneously under carbon limitation [27, 28]. A newly developed extended 16-flask RAMOS enables the investigation of up to eight conditions in biological duplicates under identical conditions, rendering additional replicates of the reference superfluous. Consequently, seven carbon sources can be investigated in a single experiment. The advantages of the extended RAMOS device are also illustrated in the Supplementary Information Fig. S1.

A schematic illustration of the in-house build 16-flask RAMOS is given in the Supplementary Information Fig. S2. The measurement technique and calculation of OTR are described in detail by Anderlei et al. [21–23]. The setup was adapted from Finger et al. [29]. For the measurement of *E. coli* cultivations, a measurement cycle of 30 min was used first [21], which was revised to 15 min after the first experiment. For *Ustilago* ssp. cultivations, a measurement cycle of 30 min was used.

## Results and discussion

### Demonstration and validation of the methodology with *E. coli*

To ensure the reliability and accuracy of the developed method, it was first validated using the model organism *E. coli*. The conclusion on the order of consumption drawn from the OTR data will be compared with the results obtained from the state-of-the-art sampling and offline analytics. Chosen for its prevalence in biotechnological applications, *E. coli* BL23(DE3) serves as a model organism for validating the presented method. To test the applicability of the method at standard cultivation parameters, *E. coli* was cultivated at 37 °C using 5 g/L each of five carbon sources. Glucose was used as the standard substrate, alongside two C5 sugars, xylose and arabinose, and two sugar alcohols, sorbitol and glycerol.

**Fig. 2:**
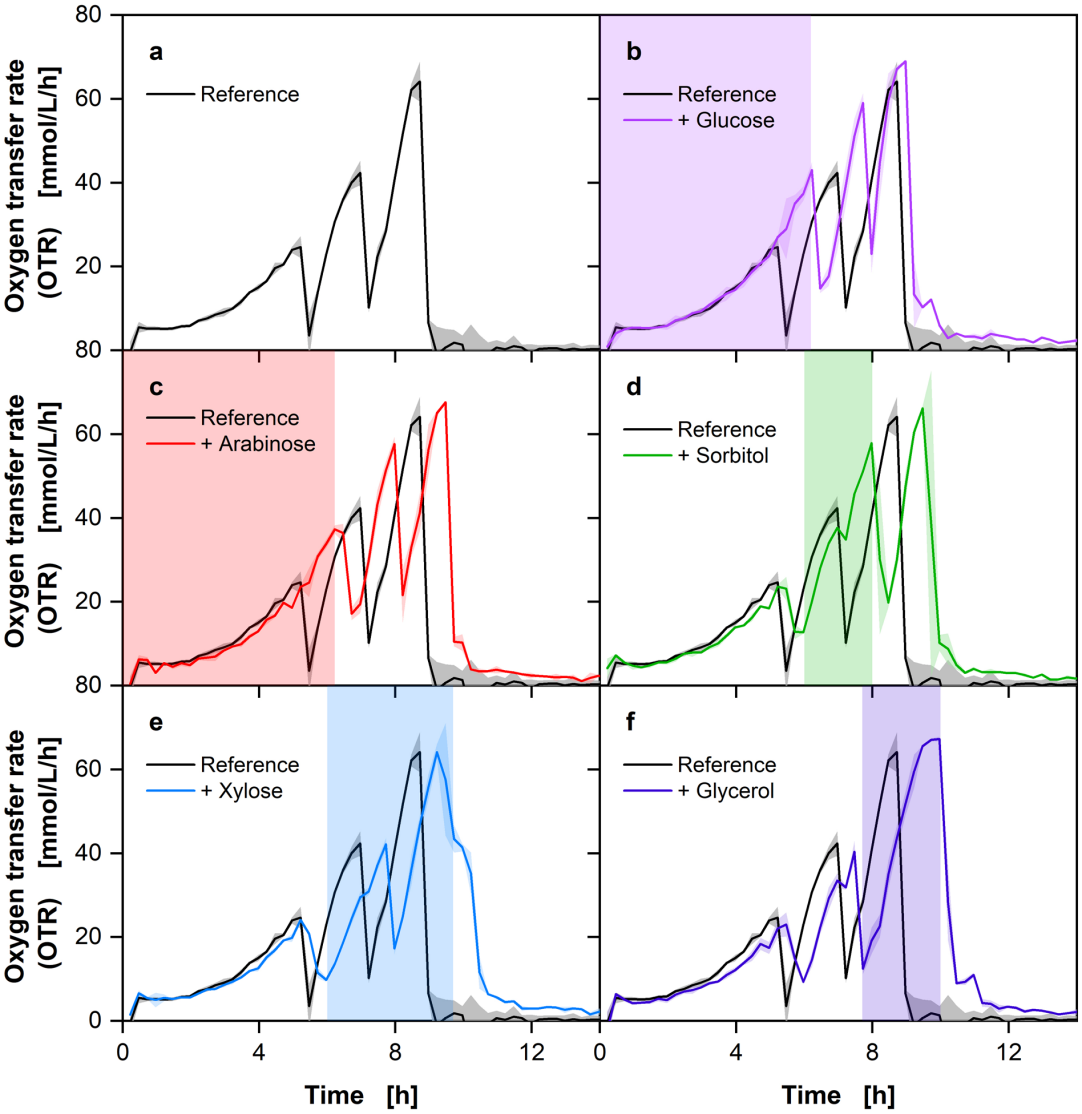
Optimized method for polyauxic growth characterization via OTR on five carbon sources with *Escherichia coli* BL23 (DE3). The reference medium contained 2.5 g/L each of the carbon sources glucose, arabinose, sorbitol, xylose, and glycerol. Additional carbon source addressed by legend was supplied with additional 2.5 g/L, resulting in a total concentration of 5 g/L. Lines represent the averages of biological duplicates and shadows indicate the minimum and maximum values of these duplicates. Estimated time of carbon source consumption is indicated by the colored vertical area. Cultivation conditions: Wilm-MOPS medium, 250 mL shake flasks, V_L_ = 10 mL, *T* = 30 °C, *n* = 350 rpm, d_0_ = 50 mm, OD_600, Start_ = 0.5 [-]

The initial trial of the developed method for polyauxic growth determination on five carbon sources with *E. coli* is depicted in the Supplementary Information Fig. S3. An exponential increase in the OTR is visible, which subsequently reached a plateau. This indicated that oxygen limitation was covering the formation of distinguishable OTR peaks, that are necessary for the developed method. The rapid and high growth observed under the standard cultivation temperature of 37 °C and the amount of carbon source provided necessitated methodological refinements. These were achieved by halving the amount of each carbon source, lowering the temperature to 30 °C, and increasing the measurement frequency of the RAMOS from every 30 min to every 15 min. The resulting measurements and distinguishable OTR peaks (Fig. 2) significantly improved the methodology’s effectiveness in subsequent experiments.

**Fig. 3:**
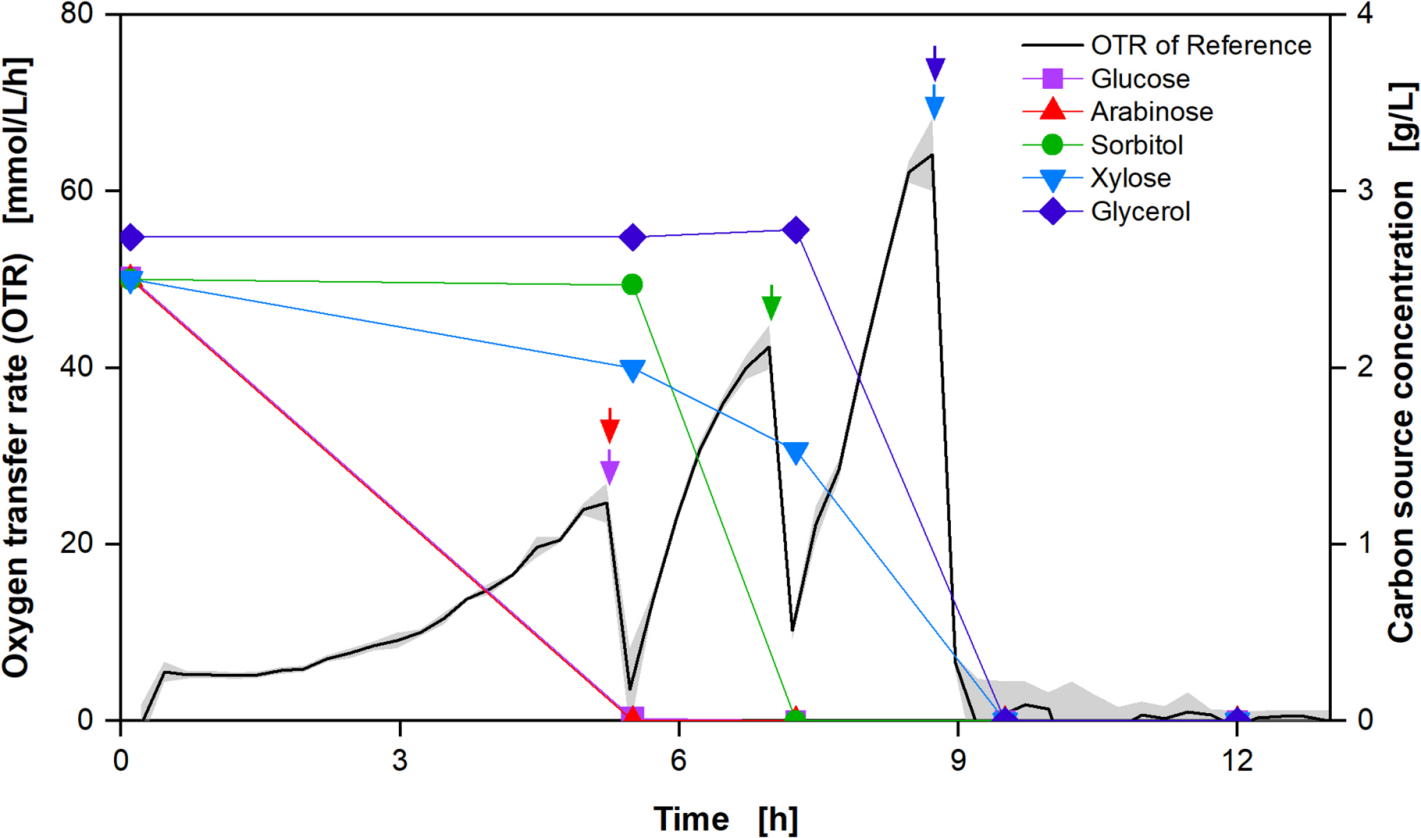
Method validation via HPLC measurement for the experiment with *Escherichia coli* presented in Fig. 2. The carbon source concentrations are plotted with the OTR of the reference cultivation. Colored arrows represent the time point where each carbon source is depleted. For cultivation details, see Fig. 2

The experiment was repeated under the revised conditions described above. The resulting data, depicted in Fig. 2, display the expected distinctive phases, indicating the order of carbon source consumption. Additionally, to validate the methodology, the samples were taken from the reference after each peak in the OTR, and the carbon source concentration was measured via HPLC (Fig. 3).

Three distinct peaks are visible in the OTR course of the reference cultivation, indicating a parallel consumption of two or more carbon sources, since with a strictly hierarchical metabolism one carbon source should result in one OTR peak. With the addition of glucose (Fig. 2b), the first peak which reaches an OTR of around 40 mmol/L/h, is considerably prolonged, compared to the reference culture. This indicates that glucose is consumed, prolonging the first OTR peak. A comparable outcome is observed with the supplementation of arabinose (Fig. 2c), which also results in a prolonged initial peak, suggesting that arabinose is also consumed within the first OTR peak. In the case of sorbitol addition (Fig. 2d), the second peak with an OTR of approximately 55 mmol/L/h is significantly prolonged compared to the reference. This suggests that sorbitol is only utilized after glucose and arabinose are depleted. The addition of xylose (Fig. 2e) shows a unique pattern. The second peak is not significantly prolonged, but rather slowed down, while the third peak, which reaches an OTR of about 60 mmol/L/h, is prolonged compared to the reference cultivation. This indicates that xylose is consumed more slowly, resulting in a delayed and prolonged third phase of oxygen consumption. The addition of glycerol (Fig. 2f) results in a significantly prolonged third peak, also reaching an OTR of about 65 mmol/L/h. This suggests that glycerol and xylose are the last carbon sources being consumed within the experiment, thus maintaining metabolic activity during the final phase of cultivation. To investigate the observed co-metabolism of glucose, arabinose, and xylose in more detail, the OTR integrals of the different peaks were compared with each other. A graphical plot of this comparison can be found in the Supplementary Information Fig. S4. From the analysis of this comparison, it can be shown that the required oxygen, which is represented by the OTR integral, is only increased in the first peak by the addition of glucose and arabinose. This leads to the conclusion that only glucose and arabinose are metabolized in the first peak, but unfortunately gives no further indication of co-metabolization. A comparison of the integrals during the addition of xylose clearly shows that both the second and the third peak are significantly prolonged. This shows that xylose is metabolized simultaneously with other carbon sources, in this example sorbitol and glycerol. These observations are consistent with the HPLC data presented in Fig. 3, which provides a comprehensive analysis of the reference cultivation. The samples were taken after each peak in the OTR to monitor the consumption of the carbon sources.

In the reference cultivation, glucose and arabinose are undetectable after the first peak, indicating their complete consumption during this phase. The formation of this single peak may be attributed to the simultaneous metabolism of the two carbon sources by *E. coli* or it could be explained by the time resolution, which may not be sufficient to capture the diauxic behavior between the two carbon sources. However, a review of the existing literature suggests that glucose and arabinose should be metabolized sequentially [30, 31]. An additional method for differentiating between the two carbon sources would be to utilize the degree of reduction in the form of the respiration quotient (RQ). It is unfortunate that the RQ is not only more susceptible to fluctuations, but also offers no advantage in differentiating between the used carbon sources arabinose and glucose, as the degree of reduction is the same. Nevertheless, it may be employed in future experiments to differentiate between carbon sources with varying degrees of reduction. Xylose exhibits a slight decline following the initial peak, indicating continuous consumption during the fermentation. This trend persists after the second peak, indicating further xylose metabolism. After the second peak, sorbitol is fully metabolized. At the end of the third peak, both xylose and glycerol are completely metabolized. The HPLC results confirm the observations made from the OTR data, thereby validating the sequential and preferential utilization of the carbon sources. The prolonged OTR peaks and corresponding HPLC data indicate that glucose and arabinose are consumed first, followed by sorbitol. Xylose is consumed the whole cultivation time in parallel and glycerol is the last carbon source being consumed by *E. coli*. The order of metabolization, which was recorded online using the newly developed methodology and offline using HPLC analysis, agrees well with the available literature. Some research suggests a dependence of the carbon source preference on the corresponding growth rate [30]. Following this hypothesis, the following metabolization sequence results from the published growth rates on the associated carbon sources: Glucose is metabolized first, followed by arabinose, xylose, sorbitol, and finally glycerol [30–33]. This sequence fits very well with our findings. Two observations do not fully agree with the literature: as already described, we can see some limitations for the presented methodology, as the literature mainly states that glucose and arabinose are metabolized sequentially. It is unfortunate that the methodology presented does not allow for the observation of this sequential metabolism, as the resolution is likely insufficient, particularly during the initial growth phase. In addition, in the experiments (Fig. 2) a parallel metabolism of xylose and other carbon sources with glucose was observed, which is also not described in the literature, but quickly becomes evident when the HPLC & OTR integral data are taken into account [30, 31].

### Demonstration of the methodology’s versatility with the fungus *U. trichophora*

**Fig. 4:**
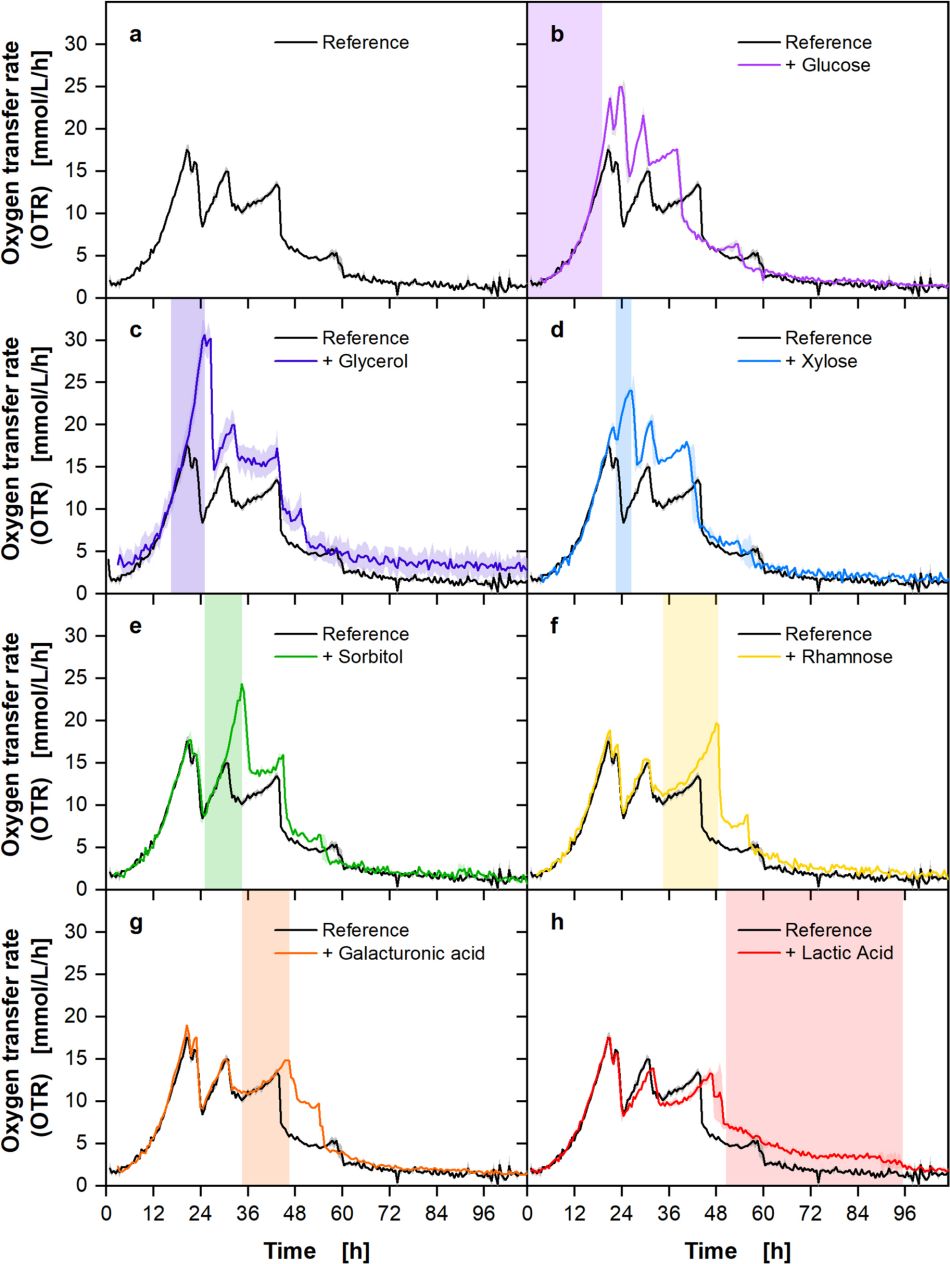
Demonstration of the versatility of polyauxic growth characterization via OTR on seven carbon sources with *Ustilago trichophora* TZ1. The reference medium contained 5 g/L each of the carbon sources glucose, glycerol, xylose, sorbitol, rhamnose, galacturonic acid, and lactic acid. Additional carbon source addressed by legend was supplied with additional 5 g/L, resulting in a total concentration of 10 g/L. Graphs were shifted along the time axis to account for slight deviations in inoculation density (Supplementary Information Table S1). Lines represent the averages of biological duplicates and shadows indicate the minimum and maximum values of these duplicates. Estimated time of carbon source consumption is indicated by the colored vertical area. Cultivation conditions: modified Verduyn medium, 250 mL shake flasks, V_L_ = 10 mL, *T* = 25 °C, *n* = 350 rpm, d_0_ = 50 mm, OD_600, Start_ = 0.3 [-]

To demonstrate the method’s versatility, the potential of the smut fungus *U. trichophora* to consecutively metabolize seven carbon sources (three sugars, two sugar alcohols, and two organic acids) was investigated. Ustilaginaceae have gained increasing interest, due to their inherent production of versatile value-added chemicals and broad substrate range [16]. *E. coli* grows approximately four times faster than *U. trichophora* [34, 35], necessitating a customization of the methodology. The reference medium contained 5 g/L of glucose, xylose, rhamnose, glycerol, sorbitol, galacturonic acid, and lactic acid, respectively. Each carbon source was individually increased to 10 g/L and cultivation was carried out at 25 °C, 5 °C below the optimal growth temperature. The profiles of the OTR curves over time are shown in Fig. 4. The period, when the investigated carbon source was consumed, is highlighted in the respective subfigure. No residual carbon source was found at the end of the cultivation via HPLC, confirming complete consumption thereof.

Five distinct peaks are visible in the OTR curve of the reference (Fig. 4a). Glucose and glycerol are consumed first, as shown by the increase of the first peak (Fig. 4b, c). When grown on additional glucose, the OTR shows a slight deviation from the reference after 17 h due to the higher slope of the curve compared to the reference. In contrast, the OTR of the culture grown on additional glycerol is congruent to the reference until the peak of the reference. This can be attributed to a slight decrease in the growth rate of *U. trichophora* when switching from glucose to glycerol [12]. It may therefore be concluded, that glucose is consumed before glycerol. Next, xylose is metabolized (Fig. 4d), followed by the consumption of sorbitol (Fig. 4e), indicated by the rise in OTR of the second and third peak, respectively. The addition of rhamnose and galacturonic acid both increased the OTR of the fourth peak (Fig. 4f–g). Hence, no clear order of consumption is visible from the OTR data. Finally, the increased lactic acid concentration prolonged the last OTR peak until 95 h, indicating that lactic acid is consumed last (Fig. 4h). For all carbon sources except for lactic acid, no deviation of the OTR curve from the reference was visible until the respective carbon source was consumed. Increased lactic acid concentrations, however, led to a delay of the preceding OTR peaks.

Limited data exist on the sequence of carbon source consumption by *Ustilago* spp., as most studies focus on glucose-based production processes. The preferences for carbon sources can, therefore, only be estimated. Generally, *Ustilago* spp. prefer glucose over xylose [36–38], galacturonic acid [39] and lactic acid [40]. Additionally, an inhibiting effect of lactic acid on growth was previously hypothesized [41]. According to the literature, the growth behavior of *U. trichophora* on rhamnose and glycerol is unusual, as no or only slow growth was reported for other *Ustilago *spp. [37, 42, 43]. However, the strain used in this study was specifically optimized for growth on glycerol via adaptive laboratory evolution, thus, efficiently utilizing glycerol for growth [12]. To our knowledge, no literature on the growth of *Ustilago* spp. on sorbitol has been published to date.

The slower growth of *U. trichophora* results in a significantly longer overall cultivation time than for *E. coli*. Therefore, almost four times more data points were measured during the cultivation of *U. trichophora*, which provides an even more precise time resolution. The adaptability of the method and the application to both fast- and slow-growing microorganisms were demonstrated. Previous studies showed, that the characteristic phases of carbon source consumption are also visible in the OTR of mammalian cell cultures [44] or the carbon dioxide transfer rate of anaerobic cultivations [45], suggesting an even broader applicability.

### Application on the next generation feedstock corn leaves with U. maydis

**Fig. 5:**
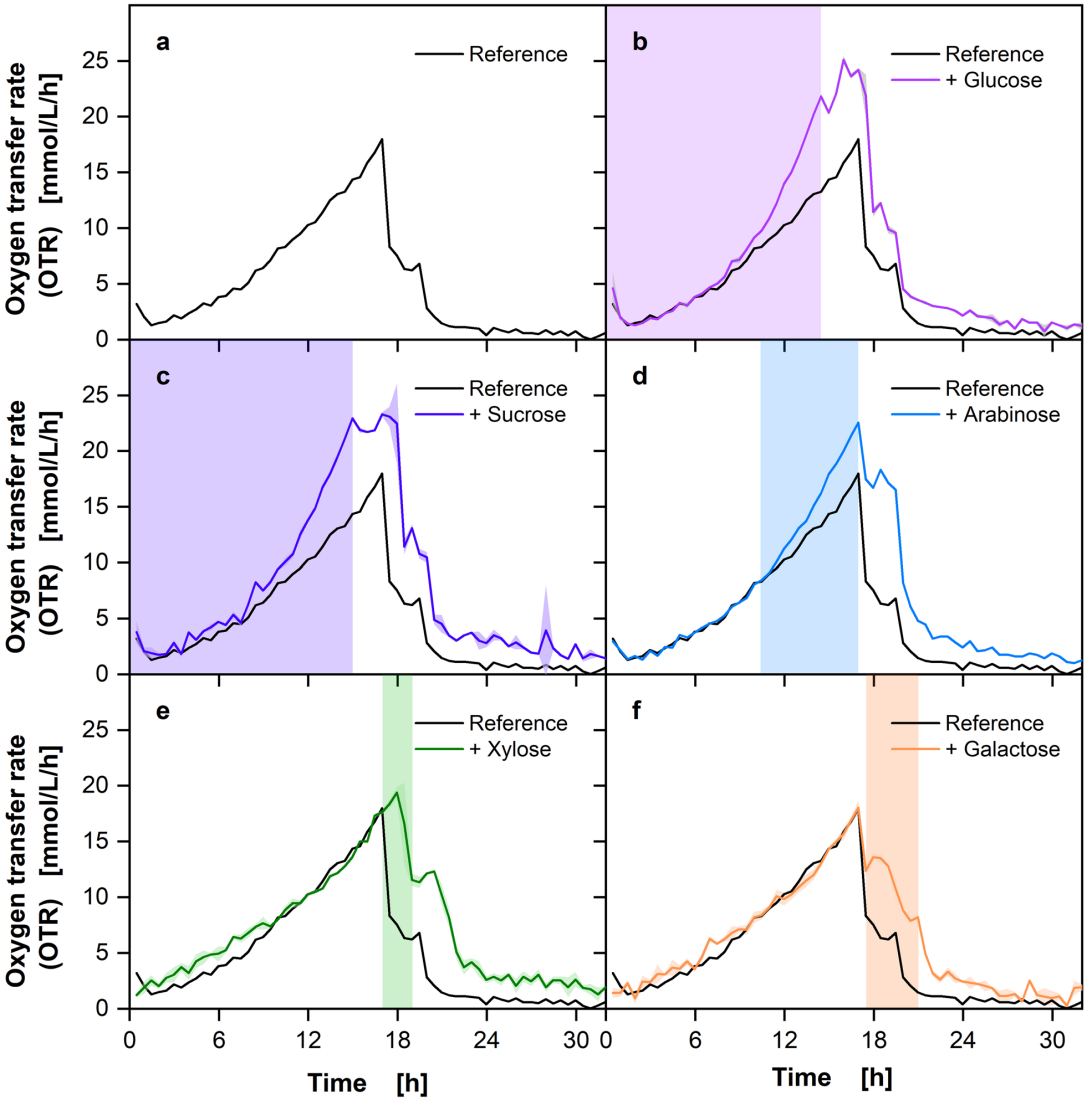
Application of polyauxic growth characterization via OTR on the crude substrate corn leaf hydrolysate with *Ustilago maydis* MB215Δcyp1Δemt1. The reference medium contained 5.4 w/v % of hydrolyzed corn leaves. Additional carbon source addressed by legend was supplied with additional 5 g/L. The graphs were shifted along the time axis to account for slight deviations in inoculation density (Supplementary Information Table S1). Lines represent the averages of biological duplicates and shadows indicate the minimum and maximum values of these duplicates. Shadows indicate the minimum and maximum values of biological duplicates. Estimated time of carbon source consumption is indicated by the colored vertical area. Cultivation conditions: modified Verduyn medium, 250 mL shake flasks, V_L_ = 10 mL, *T* = 25 °C, *n* = 350 rpm, d_0_ = 50 mm, OD_600, Start_ = 0.3 [-]

To demonstrate the practical applicability of the developed method, it was used on the plant-based feedstock hydrolyzed corn leaves. This approach not only allows to trace the sequence of metabolization but additionally identifies the carbon sources present in the complex substrate. Medium with a defined quantity of the complex substrate was used as the reference. The presumed carbon sources contained in the complex substrate were then added to the corresponding cultivation. According to the literature, the main carbon sources present in this substrate are glucose, sucrose, a disaccharide composed of glucose and fructose, arabinose, xylose, and galactose [4, 5, 46, 47]. The conditions optimized in the previous experiments with regard to the lowered temperature and the spiked carbon sources were adopted for this experiment. Therefore, a cultivation temperature of 25 °C was chosen, which is 5 °C below the normally applied growth temperature of *U. maydis* [48]. The exact carbon source concentration in the provided feedstock was unknown and the concentration of the supplemented carbon sources was set to 5 g/L. The OTR data recorded for the cultures is shown in Fig. 5.

The OTR for the reference condition (Fig. 5a) exhibits only two distinct peaks and a more linear than exponential growth pattern. This could be attributed to the sequential metabolization of different carbon sources present in the complex substrate. The necessity to switch between carbon sources and to build up the corresponding enzymes likely results in a slower, non-exponential growth pattern [9]. Upon the addition of glucose (Fig. 5b) and sucrose (Fig. 5c) to the culture, the OTR curves separate from the reference at around 8 h as the growth pattern shifts to a more exponential form. The first OTR peak is consequently formed earlier. The culture with added arabinose (Fig. 5d) exhibited a later peak separation from the reference at approximately 12 h, suggesting that arabinose is consumed by the organism subsequently to glucose and fructose (derived from sucrose). The addition of xylose (Fig. 5e) prolonged the first peak without separation of the curves, indicating that this peak in the reference represents the consumption of xylose. The addition of galactose (Fig. 5f) resulted in the formation of an additional peak at approximately 18 h, indicating that the galactose concentration in the complex carbon source is low, and does not lead to peak formation in the reference cultivation.

In summary, the consumption pattern of sugars in the complex substrate appears as follows: glucose and sucrose are at least partially consumed in parallel, as the cells break down sucrose into the individual components glucose and fructose [49]. The next carbon sources that have been consumed are arabinose and then xylose. Following the consumption of xylose, galactose is also consumed, although it appears that there are no substantial quantities of galactose present in the reference. Additionally, a final peak forms at approximately 19.5 h in the reference, which cannot be identified by the tested carbon sources. This peak likely represents the consumption of acetate, based on the small amount present, due to acetate buffer usage in the hydrolysis process and the ability of *U. maydis* to metabolize it [4, 43]. The data collected are in good agreement with the current literature, regarding the metabolic properties of *U. maydis*, as we observed that all carbon sources were metabolized [34, 50]. Regarding the order of metabolism, a strong preference for glucose and fructose can also be identified in the literature [49]. Again, by comparing the growth rates on the different carbon sources, it is visible that xylose, arabinose, and galactose are not preferred carbon sources as they result in a reduced growth rate [34, 49, 50]. In summary, the method was able to confirm the sugars contained in the raw substrate and provide their order of metabolization. This is particularly important for work with crude substrates such as plant material, as HPLC analysis is often difficult or impossible with such complex feedstocks [51]. It should be noted, however, that the method presented herein is not without limitations when complex feedstocks are employed. To apply this method, it is necessary to have at least some knowledge of the carbon sources present in the feedstock. Furthermore, the concentration of the feedstock can only be adjusted to a limited extent, which could result in oxygen limitations or an inadequate resolution. However, this could be mitigated by reducing the cultivation temperature. Another challenge is the presence of highly variable carbon sources concentrations within a feedstock, which could result in very small concentrations being undetected, when carbon sources with high concentrations are present. Nevertheless, these low concentrations would then be of secondary importance for process control.

## Conclusion and outlook

The methodology developed in this study provides substantial insights into the metabolic behavior of aerobic microorganisms when confronted with diverse metabolizable carbon sources. The results obtained using this online determination method are in close agreement with those obtained from offline sampling and subsequent HPLC measurement, thereby demonstrating the accuracy and reliability of the method. Moreover, this method showed its versatility by being suitable for both fast-growing bacteria and slow-growing fungi, thereby making it broadly applicable in studies regarding microbial substrate preferences. Previous studies showed, that the characteristic phases of carbon source consumption are also visible in the OTR of mammalian cell cultures [44] or the carbon dioxide transfer rate of anaerobic cultivations [45], suggesting an even broader applicability with a slight adaptation of the method.

In addition, the method has been successfully applied to a renewable raw material utilizing corn leaf hydrolysate, thus enabling the analysis of metabolizable components of corn leaf hydrolysate and their order of metabolization by *U. maydis*. In general, it should be feasible to analyze other complex feedstocks in a similar manner, although the same limitations will likely apply. The complex carbon source utilized must allow for sufficient growth of the organism, must not be excessively concentrated, and a preliminary estimation of the potential carbon sources present is essential.

With the aforementioned limitations the developed method could be applied to a broader range of microorganisms and more renewable raw materials. To enhance the screening throughput, the same method could also be transferred to microtiter-scale, using the newly established µTOM [52]. Furthermore, the data already collected, as well as future data, could be used together with the respective growth rates in a mathematical model to make even more precise predictions about the sequence of metabolization [53]. In addition, an application to other essential nutrients like the nitrogen sources would be conceivable, as these also influence the respiration activities of the culture.

## Supplementary Information

Below is the link to the electronic supplementary material.

Supplementary file1 (PDF 454 KB)

## Data Availability

Data is provided within the manuscript or supplementary information files.
