# Supplementary material for: Sampling-free investigation of microbial carbon source preferences on renewable feedstocks via online monitoring of oxygen transfer rate
Source: Bioprocess Biosyst Eng. 2024 Dec 16;48(3):413–25. doi: 10.1007/s00449-024-03117-x (PMC11865135; doi:10.1007/s00449-024-03117-x)
Supplement: Supplementary file 1 — Supplementary file1 (PDF 454 KB) [file 449_2024_3117_MOESM1_ESM.pdf]

Sampling-free investigation of microbial carbon source preferences on renewable feedstocks via online monitoring of oxygen transfer rate

In Bioprocess and Biosystems Engineering

Luca Antonia Grebe[1, †], Paul Richter[1, 2, †], Torben Altenkirch[1], Marcel Mann[1, 2], Markus Müller[1], Jochen Büchs[1, 2], Jørgen Barsett Magnus[1, 2, *]

[1]AVT - Biochemical Engineering, RWTH Aachen University, Forckenbeckstraße 51, 52074 Aachen, Germany

[2] Bioeconomy Science Center (BioSC), 52425 Jülich, Germany

[†]Authors contributed equally to this work

* Corresponding author:
Prof. Dr.-Ing. Jørgen Magnus (jorgen.magnus@avt.rwth-aachen.de), AVT – Biochemical Engineering, RWTH Aachen University, Forckenbeckstraße 51, 52074 Aachen, Germany, Phone: +492418023569

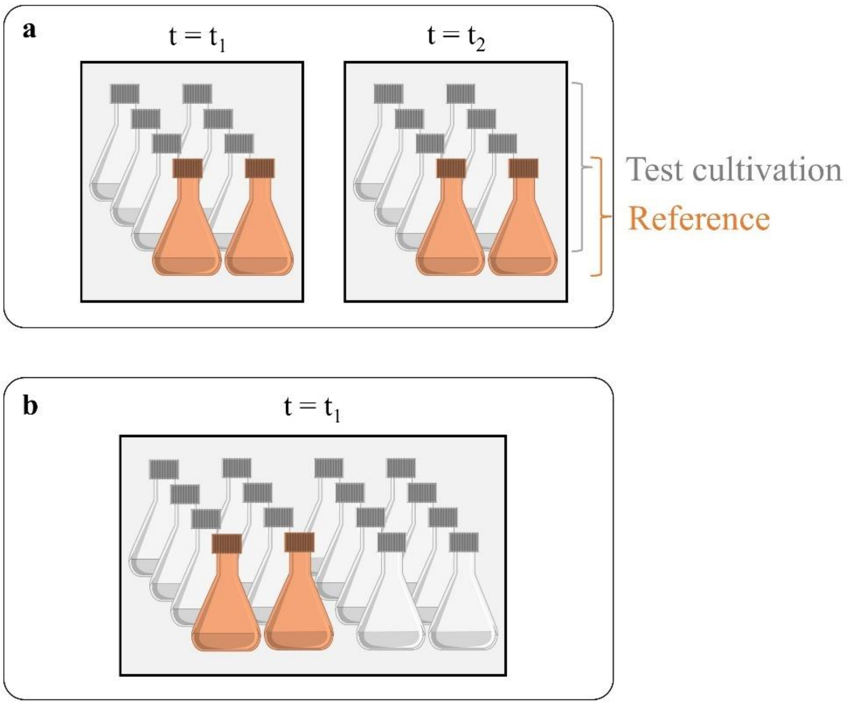

Fig. S1: Schematic illustration of the advantages of the 16-flask RAMOS device compared to the standard 8-flask RAMOS device. **a** Consecutive investigation of six carbon sources. **b** Simultaneous investigation of seven carbon sources

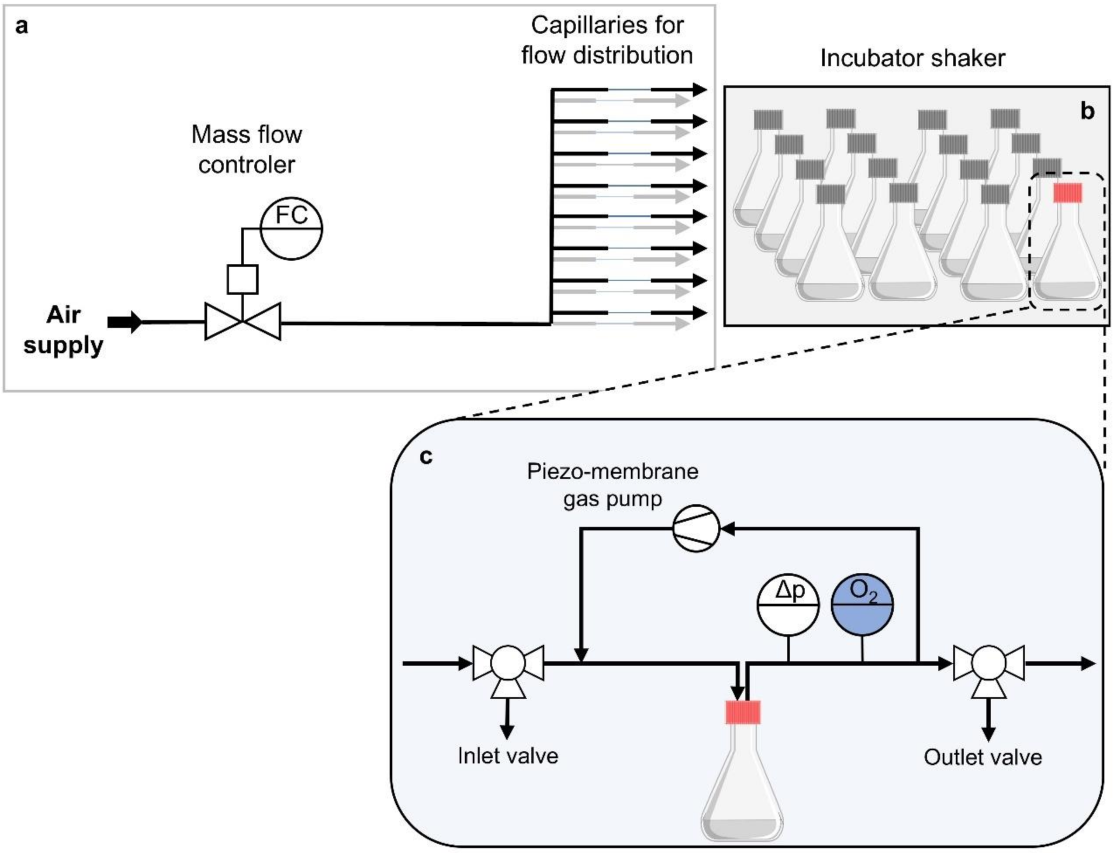

Fig. S2: Schematic illustration of the 16-flask RAMOS. **a** Air supply controlled by thermal mass flow controller and capillaries to ensure even gas distribution to all 16 flasks. **b** Temperature-controlled shaker, containing 16 measurement flasks. **c** Measurement loop of one shake flask with in- and outlet valve, piezo-membrane gas pump, pressure sensor, and oxygen sensor. The measurement technique and calculation of OTR are described in detail by Anderlei et al. [1, 2]. The setup was adapted from Finger et al. [3]

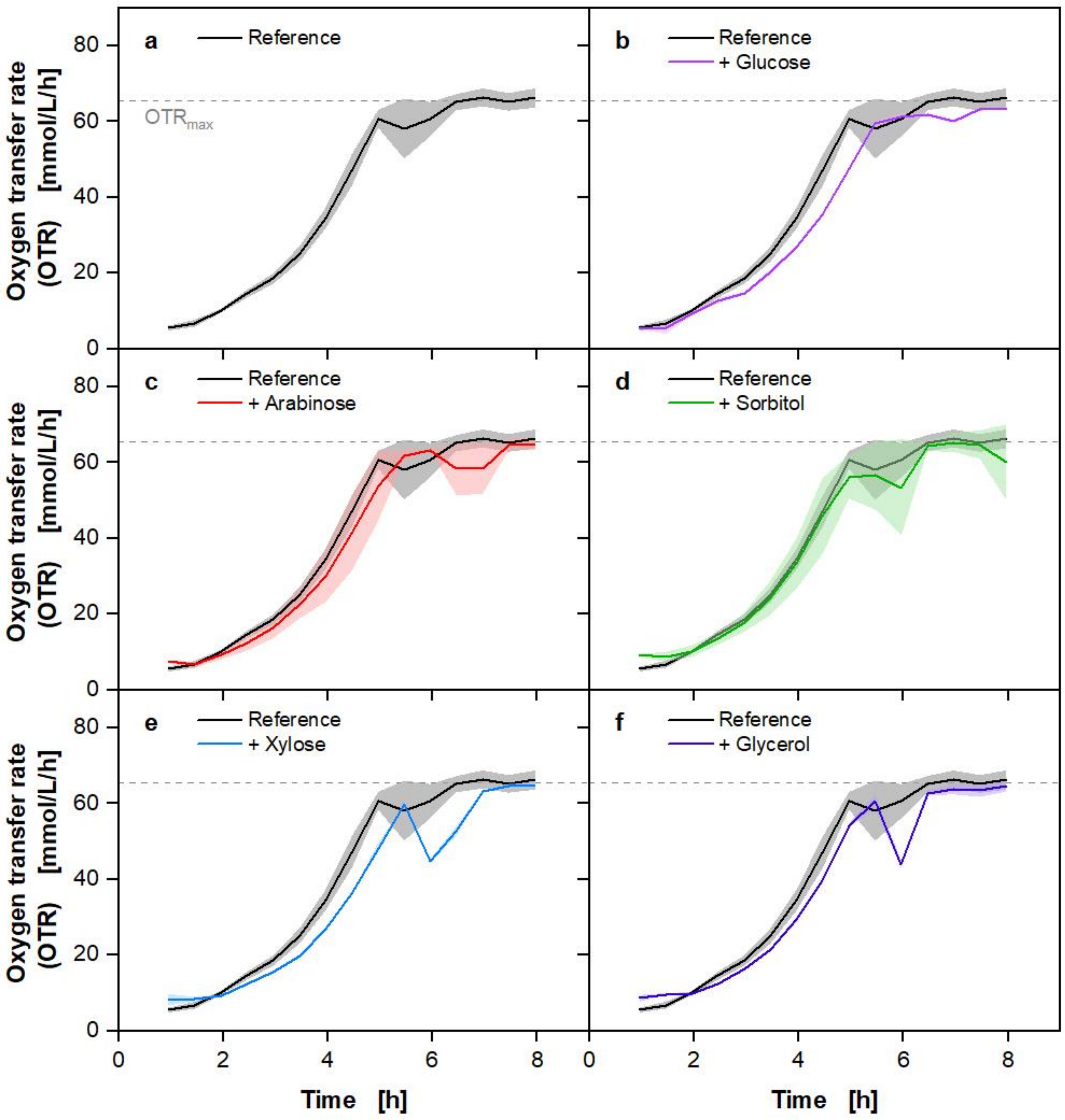

Fig. S3: Initial trial of polyauxic growth characterization via OTR on five carbon sources with *Escherichia coli* BL23(DE3). The reference medium contained 5 g/L each of the carbon sources glucose, arabinose, glycerol, xylose, and sorbitol. Additional carbon source addressed by legend was supplied with additional 5 g/L, resulting in a total concentration of 10 g/L. Lines represent the averages of biological duplicates and shadows indicate the minimum and maximum values of these duplicates. Cultivation conditions: Wilm-MOPS medium, 250 mL shake flasks, V_L = 10 mL, T = 37 °C, n = 350 rpm, d_0 = 50 mm, $OD_{600, Start}$ = 0.5 [-]

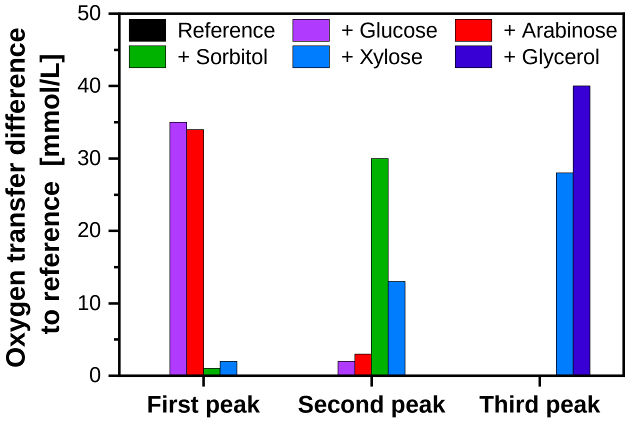

Fig. S4: Graphical presentation of the consumed oxygen, derived from the OTR integral, in the three OTR peaks. To enable a clearer comparison, the OTR integrals are shown as the difference to the reference. The corresponding cultivation data can be found in Fig. 2.

Table S1: Time shift of graphs in regard to the reference in Fig. 4 and Fig. 5

Investigated carbon source	Timeshift [h]
**Fig. 4:** Demonstration of versatility with *U. trichophora*	
Glucose	2.0
Galacturonic acid	1.5
Xylose	2.0
Glycerol	2.5
Rhamnose	0.0
Lactic acid	0.0
Sorbitol	1.0
**Fig. 5:** Application on the crude substrate corn leaf hydrolysate with *U. maydis* MB215Δcyp1Δemt1	
Glucose	2.0
Sucrose	0.0
Arabinose	0.0
Xylose	2.0
Galactose	2.0

References

1. Anderlei T, Büchs J (2001) Device for sterile online measurement of the oxygen transfer rate in shaking flasks. Biochemical Engineering Journal 7:157–162. https://doi.org/10.1016/S1369-703X(00)00116-9
2. Anderlei T, Zang W, Papaspyrou M et al. (2004) Online respiration activity measurement (OTR, CTR, RQ) in shake flasks. Biochemical Engineering Journal 17:187–194. https://doi.org/10.1016/S1369-703X(03)00181-5
3. Finger M, Schröder E, Berg C et al. (2023) Toward standardized solid medium cultivations: Online microbial monitoring based on respiration activity. Biotechnol J 18:e2200627. https://doi.org/10.1002/biot.202200627
